# Antimicrobial susceptibility of gram-negative strains isolated from bloodstream infections in China: Results from the study for monitoring antimicrobial resistance trends (SMART) 2018–2020

**DOI:** 10.1017/S0950268824001286

**Published:** 2025-03-21

**Authors:** Yili Chen, Pingjuan Liu, Huayin Li, Wenxiang Huang, Chunxia Yang, Mei Kang, Xiaofeng Jiang, Bin Shan, Hong He, Fupin Hu, Pengcheng Li, Yingchun Xu, Kang Liao

**Affiliations:** 1 Department of Laboratory Medicine, The First Affiliated Hospital, Sun Yat-Sen University, Guangzhou, China; 2 Division of Microbiology, Zhongshan Hospital of Fudan University, Shanghai, China; 3 Division of Microbiology, The First Affiliated Hospital of Chongqing Medical University, Chongqing, China; 4 Department of Clinical Laboratory, Beijing Chao-Yang Hospital, Beijing, China; 5 Department of Laboratory Medicine, West China School of Medicine, West China Hospital of Sichuan University, Chengdu, China; 6 Department of Clinical Laboratory, The Fourth Affiliated Hospital of Harbin Medical University, Harbin, China; 7 Department of Clinical Laboratory, First Affiliated Hospital of Kunming Medical University, Kunming, China; 8 Department of Clinical Laboratory, The Affiliated Hospital of Qingdao University, Qingdao, China; 9 Institute of Antibiotics, Huashan Hospital, Fudan University, Shanghai, China; 10 V&I, Global Medical & Scientific Affairs, MSD China, Shanghai, China; 11 Division of Microbiology, Peking Union Medical College Hospital, Peking Union Medical College, Chinese Academy of Medical Sciences, Beijing, China

**Keywords:** bloodstream infection, gram-negative bacteria, SMART, carbapenem resistance, ESBL

## Abstract

The study aims were to present in vitro susceptibilities of clinical isolates from Gram-negative bacteria bloodstream infections (GNBSI) collected in China. GNBSI isolates were collected from 18 tertiary hospitals in 7 regions of China from 2018 to 2020. Minimum inhibitory concentrations were assessed using a Trek Diagnostic System. Susceptibility was determined using CLSI broth microdilution, and breakpoints were interpreted using CLSI M100 (2021). A total of 1,815 GNBSI strains were collected, with *E. coli* (42.4%) and *Klebsiella pneumoniae* (28.6%) being the most prevalent species, followed by *P. aeruginosa* (6.7%). Susceptibility analyses revealed low susceptibilities (<40%) of ESBL-producing *E. coli* and *K. pneumonia* to third-/fourth-generation cephalosporins, monobactamases, and fluoroquinolones. High susceptibilities to colistin (95.0%) and amikacin (81.3%) were found for *K. pneumoniae*, while *Acinetobacter baumannii* exhibited a high susceptibility (99.2%) to colistin but a low susceptibility to other antimicrobials (<27.5%). Isolates from ICUs displayed lower drug susceptibility rates of *K. pneumoniae* and *A. baumannii* than isolates from non-ICUs (all *P* < 0.05). Carbapenem-resistant and ESBL-producing *K. pneumoniae* detection was different across regions (both *P* < 0.05). *E. coli* and *K. pneumoniae* were major contributors to GNBSI, while *A. baumannii* exhibited severe drug resistance in isolates obtained from ICU departments.

## Introduction

Bloodstream infection (BSI) is a global public health problem associated with increased mortality, hospitalization time, and healthcare costs [1, 2]. Inappropriate use of antibiotics for the treatment of BSI has been shown to be independently associated with an increased risk of death [3], highlighting the crucial importance of proper antibiotic use in the treatment and prognosis of a BSI.

According to the Blood Bacterial Resistant Investigation Collaborative System (BRICS) surveillance report from China, Gram-negative bacteria (GNB) accounted for 70.5% of the collected blood bacterial strains between 2014 and 2019 [4]. BSI caused by GNB is characterized by rapid disease progression and a severe systemic inflammatory response, with a high mortality rate of 12% to 38% [5]. The prevalence of Gram-negative resistant strains varies according to the hospital type and the regional economic development level and has shown a decreasing trend since the initiation of special national antimicrobial management activity by the Chinese government in 2012 [4]. However, empirical treatment for BSI still relies heavily on carbapenems but the increasing detection rate of carbapenem-resistant *Klebsiella pneumoniae* (CRKP) poses a significant challenge to the treatment of BSI [6, 7].

Currently used automated antimicrobial susceptibility testing methods have a long reporting cycle for blood culture results. Timely and appropriate empirical antimicrobial therapy is a key factor in clinical prognosis [8]. Therefore, understanding the epidemiology and bacterial resistance data of GNB in BSI will provide a reference for the best empirical antimicrobial treatment. Furthermore, the results will highlight the changing trends in bacterial resistance and guide the rational clinical use of antimicrobial drugs and the formulation of prevention and control strategies.

The Study for Monitoring Antimicrobial Resistance Trends (SMART) global surveillance programme is a comprehensive initiative aimed at monitoring and analyzing the trends of antimicrobial resistance worldwide. In the present study, we present the distribution and in vitro susceptibility results of antimicrobials against GNB isolates submitted to the SMART programme between 2018 and 2020 by clinical laboratories in China, focussing on isolates from BSI.

## Materials and methods

### Sampling strategy and inclusion criteria of BSI isolates

From 2018 to 2020, 18 tertiary hospitals across 7 regions of China participated in the SMART global surveillance programme and were each requested to collect consecutively up to 50 GNB isolates obtained from the central venous system (CVS) per year from patients with clinical and laboratory confirmed BSI. A comprehensive list of the participating hospitals and their corresponding regions are shown in Supplementary Table 1.

All bacterial strains originated from residual samples used in clinical diagnosis without prior antibiotic treatment or from their subcultures. Only the initial isolate of each species per patient was considered eligible throughout the entire study duration. The isolates underwent initial identification using the procedures established at the local hospital. They were then transferred to the clinical microbiology laboratory of Peking Union Medical College Hospital for re-identification using MALDI TOF MS (Vitek MS, BioMérieux, France) and subsequent antimicrobial susceptibility testing. Approval for the study protocols (Ethics Number: S-K238) was obtained from the Human Research Ethics Committee of our hospital.

### Antimicrobial susceptibility testing

Minimum inhibitory concentrations (MICs) were determined by the Clinical and Laboratory Standards Institute (CLSI) reference broth microdilution method using custom-made dehydrated panels manufactured by TREK Diagnostic Systems (Thermo Fisher Scientific, Oakwood Village, OH, USA). CLSI M100 (2021) breakpoints were used for all drugs [9], with the exception of colistin, for which the European Committee on Antimicrobial Susceptibility Testing (EUCAST) susceptibility breakpoint was used [10]. Carbapenem-resistant (CR) strains were characterized as organisms that exhibited resistance to drugs within the carbapenem class. The isolates were tested to determine whether they possessed an extended-spectrum β-lactamase (ESBL) phenotype, which was determined by a ceftriaxone or ceftazidime MIC ≥ 2 mg/L. The presence of ESBL-positive strains was confirmed using a clavulanic acid-based combination test protocol that adhered to the methodology outlined by the CLSI [11].

### Statistical analysis

The data were analyzed and visualized using R (ver. 4.2.0). To evaluate differences between groups, chi-squared or Fisher’s exact tests were initially conducted, followed by a post hoc test with the Bonferroni correction applied and adjusted standardized residuals. Statistical significance was defined as a *P* value <0.05. We employed the chi-squared test for trend to assess whether there were statistically significant changes in bacterial proportions over time.

## Results

### General distribution of GNB from 2018 to 2020

A total of 1,815 strains of GNB from BSI were collected over a 3-year period. The majority of strains were collected in 2018 (*n* = 831) and 2019 (*n* = 784), accounting for 89.0% of the total, while the remaining 11.0% were collected in 2020. The most common species identified were *E. coli* (42.4%) and *K. pneumoniae* (28.6%), followed by *P. aeruginosa* (6.7%) and *Acinetobacter baumannii* (6.6%). Additionally, a significant increasing trend in the proportion of *E. coli* over the years was observed (*P* = 0.001), while a notable decreasing trend in the proportions of *A. baumannii* (*P* < 0.001) and *K. pneumoniae* (*P* = 0.037) was noted annually (Table 1).

**Table 1.** Distribution of 1815 isolates of GNB in 2018, 2019, and 2020

	2018	2019	2020	*P*-value for trend	Total
Sum	831 (45.8)	784 (43.2)	200 (11.0)		1,815 (100.0)
*Escherichia coli*	324 (39.0)	342 (43.6)	104 (52.0)	**0.001**	770 (42.4)
*Klebsiella pneumoniae*	251 (30.2)	220 (28.1)	48 (24.0)	0.080	519 (28.6)
*Pseudomonas aeruginosa*	58 (7.0)	47 (6.0)	16 (8.0)	0.993	121 (6.7)
*Acinetobacter baumannii*	77 (9.3)	35 (4.5)	8 (4.0)	**< 0.001**	120 (6.6)
*Enterobacter cloacae*	29 (3.5)	31 (4.0)	1 (0.5)	0.186	61 (3.4)
*Klebsiella aerogenes*	18 (2.2)	12 (1.5)	0 (0.0)	**0.037**	30 (1.7)
*Klebsiella oxytoca*	7 (0.8)	21 (2.7)	1 (0.5)	0.253	29 (1.6)
*Serratia marcescens*	14 (1.7)	9 (1.1)	1 (0.5)	0.153	24 (1.3)
Others	53 (6.4)	67 (8.5)	21 (10.5)	**0.026**	141 (7.8)

### Distribution characteristics of GNB strains in different departments

A total of 337 GNB strains (18.6%) were isolated from ICUs. The proportion of *A. baumannii* strains in the ICU was approximately four times higher than in non-ICU settings (17.5% vs. 4.1%), while the proportion of *E. coli* strains in the ICU was less than half of that in non-ICU settings (22.8% vs. 47.0%). The distribution of GNB in internal medicine (842 strains, 46.4%) was similar to that in surgery (579 strains, 31.9%) (Supplementary Figure 1).

### Distribution characteristics of GNB strains in different age groups and regions

The distribution of GNB strains in different age groups revealed that the proportions of *P. aeruginosa* (13.7%) and *A. baumannii* (10.5%) were higher in children and adolescents aged 0–17 years compared to those aged 18–64 years (*P. aeruginosa*: 7.2%; *A. baumannii*: 6.9%) and ≥ 65 years (*P. aeruginosa*: 5.0%; *A. baumannii*: 5.7%). In contrast, the proportion of *E. coli* (24.2%) was lower in the 0–17 years age group compared to the other two age groups (18–64 years: 41.9%; ≥65 years: 45.6%). The proportion of *K. pneumoniae* was similar across all three age groups (29.5%, 29.6%, and 27.1%, respectively) (Supplementary Figure 2).

In terms of regional distribution, the highest numbers of GNB strains were collected in the East (non-Jiangzhe Area), comprising approximately one-third of all strains (*n* = 633). In this region, the proportion of *A. baumannii* was remarkably high at 22.7%, ranking second after *E. coli* (30.0%). This represents the highest proportion of *A. baumannii* among all regions. In the East (Jiangzhe Area) (*n* = 228), *K. pneumoniae* was the most common GNB isolated. It accounted for 44.3% of all cases in this region, which was the highest proportion compared to other regions. Notably, in the Northeast (*n* = 197), the combined proportion of *P. aeruginosa* and *A. baumannii* was only 4%, and lower than that of other species such as *Enterobacter cloacae* (4.6%) and *Klebsiella oxytoca* (5.1%) (Supplementary Figure 3).

### Susceptibility analysis of main GNB to common antimicrobials

*E. coli* strains exhibited notable in vitro susceptibility, with amikacin (98.3%), carbapenems (95.5–97.7%), colistin (96.8%), and piperacillin-tazobactam (90.7%) all surpassing 90% susceptibility. For *K. pneumoniae*, colistin (95.0%) and amikacin (81.3%) were the top two ranked antibiotics in terms of susceptibility. Carbapenems showed moderate susceptibility rates ranging from 72.1% to 74.0%, while third-/fourth-generation cephalosporins, levofloxacin, and piperacillin-tazobactam had susceptibility rates of 50.7–57.8%. *P. aeruginosa* displayed favourable in vitro susceptibility to aminoglycosides (>95%), with third-/fourth-generation cephalosporins, fluoroquinolones, and colistin ranging from 78.5% to 84.3%. Meropenem and piperacillin-tazobactam exhibited susceptibility rates of 77.7% and 70.3%, respectively. *A. baumannii* displayed excellent susceptibility to colistin (99.2%) but limited susceptibility (<27.5%) to other antimicrobials, including imipenem and meropenem (both 20.0%). *E. cloacae* had a high susceptibility to amikacin (96.7%) and meropenem (95.1%) but limited susceptibility to ceftriaxone (47.5%) (Table 2).

**Table 2.** Annual susceptibility rates of common antimicrobials against Gram-negative bacilli

	*E. coli* (*n* = 770)				*Klebsiella pneumoniae* (*n* = 519)				*P. aeruginosa* (*n* = 121)				*Acinetobacter baumannii* (*n* = 120)				*Enterobacter cloacae* (*n* = 61)*		
	Overall	2018	2019	2020	Overall	2018	2019	2020	Overall	2018	2019	2020	Overall	2018	2019	2020	Overall	2018	2019
AMK	98.3	97.8	98.5	99.0	81.3	81.3	80.0	87.5	95.9	91.4	100.0	100.0	27.5	24.7	31.4	37.5	96.7	96.6	100.0
FOX	77.9	76.2	80.7	74.0	62.4	63.0	59.1	75.0	N	N	N	N	N	N	N	N	N	N	6.5
CRO	41.4	40.4	43.3	38.5	50.7	50.6	48.2	62.5	N	N	N	N	N	N	N	N	47.5	44.8	51.6
CAZ	63.5	63.6	64.3	60.6	57.8	58.6	54.1	70.8	78.5	84.5	78.7	56.3	22.5	20.8	25.7	25.0	59.0	69.0	51.6
FEP	48.7	46.0	48.8	56.7	56.1	54.6	55.9	64.6	84.3	86.2	85.1	75.0	20.8	18.2	22.9	37.5	77.1	75.9	80.7
ETP	95.5	94.1	96.2	97.1	72.1	73.3	68.6	81.3	N	N	N	N	N	N	N	N	78.7	82.8	77.4
IPM	97.3	96.6	97.7	98.1	73.8	74.1	71.8	81.3	63.6	63.8	68.1	50.0	20.0	18.2	20.0	37.5	88.5	86.2	93.6
MEM	97.7	96.9	98.3	98.1	74.0	73.3	72.7	83.3	77.7	75.9	76.6	87.5	20.0	18.2	20.0	37.5	95.1	93.1	100.0
ATM	54.2	53.4	54.1	56.7	57.2	56.2	55.9	68.8	71.9	75.9	70.2	62.5	N	N	N	N	60.7	65.5	58.1
TZP	90.7	92.6	88.0	93.3	63.8	66.1	57.3	81.3	70.3	77.6	61.7	68.8	20.0	18.2	20.0	37.5	65.6	69.0	61.3
LVX	46.4	47.5	47.1	40.4	64.7	64.5	61.8	79.2	84.3	82.8	85.1	87.5	21.7	19.5	22.9	37.5	85.3	82.8	87.1
COL	96.8	96.3	97.7	95.2	95.0	96.4	93.2	95.8	82.6	82.8	76.6	100.0	99.2	98.7	100.0	100.0	78.7	86.2	74.2

Note: *: In 2020, only one isolate of *E. cloacae* was detected; therefore, susceptibility data for 2020 is not presented. Data are given as percentages.

Abbreviations: AMK, amikacin; ATM, aztreonam; CAZ, ceftazidime; COL, colistin; CRO, ceftriaxone; ETP, ertapenem; FEP, cefepime; FOX, cefoxitin; IPM, imipenem; LVX, levofloxacin; MEM, meropenem; N, Not tested; TZP, piperacillin-tazobactam.

On an annual basis, the susceptibility rates of *K. pneumoniae* to carbapenems and piperacillin-tazobactam in 2020 were higher than those in 2018 and 2019, exceeding 80%. In contrast, the susceptibility rates of *P. aeruginosa* to ceftazidime and imipenem in 2020 showed a decrease of more than 10% compared to the previous 2 years, while the susceptibility rates to meropenem and colistin increased by over 10%. Additionally, the susceptibility rates of *A. baumannii* to carbapenems, piperacillin-tazobactam, and levofloxacin in 2020 improved by more than 15% compared to the preceding years. Over the 3-year period, the annual susceptibility rates of common antibiotics to *E. coli* remained relatively stable (Table 2).

### Susceptibility profiles of E. coli and K. pneumoniae: ESBL and carbapenem resistance

We further evaluated the susceptibility profiles of *E. coli* and *K. pneumoniae*, categorized based on the presence or absence of ESBL and CR strains. Overall, most antimicrobial agents demonstrated a markedly high susceptibility profile against *E. coli* ESBL− isolates (*n* = 319) and *K. pneumoniae* ESBL isolates (*n* = 263). *E. coli* ESBL+ (*n* = 426), and *K. pneumoniae* ESBL+ (*n* = 115) strains exhibited substantial susceptibility to carbapenems, with sensitivity rates ranging from 96.5% to 100.0%, underscoring the efficacy of these antibiotics against ESBL-producing Enterobacteriaceae. CR-*E. coli* (n = 26) exhibited limited susceptibility to most antibiotics (3.8–30.8%), with the highest sensitivities being found for amikacin (92.3%) and colistin (88.5%). Furthermore, CR-*K. pneumoniae* (*n* = 141) displayed notable resistance, with restricted sensitivity found for various antibiotics, except for colistin where a substantial susceptibility was predominantly evident (93.6%) (Table 3).

**Table 3.** Susceptibility rates of common antimicrobials against ESBL−, ESBL+, and CR-*E. coli* and *Klebsiella pneumoniae*

	*E. coli* ESBL− (*n* = 319)	*E. coli* ESBL+ (*n* = 426)	CR-*E. coli* (*n* = 26)	*K. pneumoniae* ESBL− (*n* = 263)	*K. pneumoniae* ESBL+ (*n* = 115)	CR-*K. pneumoniae* (*n* = 141)
AMK	100.0	97.4	92.3	99.2	96.5	35.5
FOX	91.2	72.3	3.8	91.6	70.4	1.4
CRO	100.0	0.0	3.8	100.0	0.0	0.0
CAZ	99.1	40.1	11.5	98.9	33.9	0.7
FEP	99.7	12.9	11.5	100.0	22.6	1.4
ETP	99.7	97.4	11.5	100.0	96.5	0.0
IPM	99.7	99.8	23.1	100.0	98.3	5.0
MEM	100.0	100.0	30.8	100.0	99.1	5.0
ATM	100.0	21.4	30.8	100.0	25.2	3.5
TZP	97.2	89.9	23.1	95.8	67.0	1.4
LVX	68.3	31.9	15.4	95.1	66.1	7.1
COL	97.8	96.2	88.5	96.6	93.0	93.6

Note: Data are presented as percentages.

Abbreviations: AMK, amikacin; ATM, aztreonam; CAZ, ceftazidime; COL, colistin; CR, carbapenem-resistant; CRO, ceftriaxone; ETP, ertapenem; FEP, cefepime; FOX, cefoxitin; IPM, imipenem; LVX, levofloxacin; MEM, meropenem; TZP, piperacillin-tazobactam; *E. coli* ESBL+, ESBL-producing *Escherichia coli*; *E. coli* ESBL−, non-ESBL-producing *E. coli*; *K. pneumoniae* ESBL+, ESBL-producing *K. pneumoniae; K. pneumoniae* ESBL−, non-ESBL-producing *K. pneumoniae.*

### Comparison of internal medicine vs. surgery, ICU vs. non-ICU for the susceptibility of the four major GNB to antimicrobials

**Figure 1.**
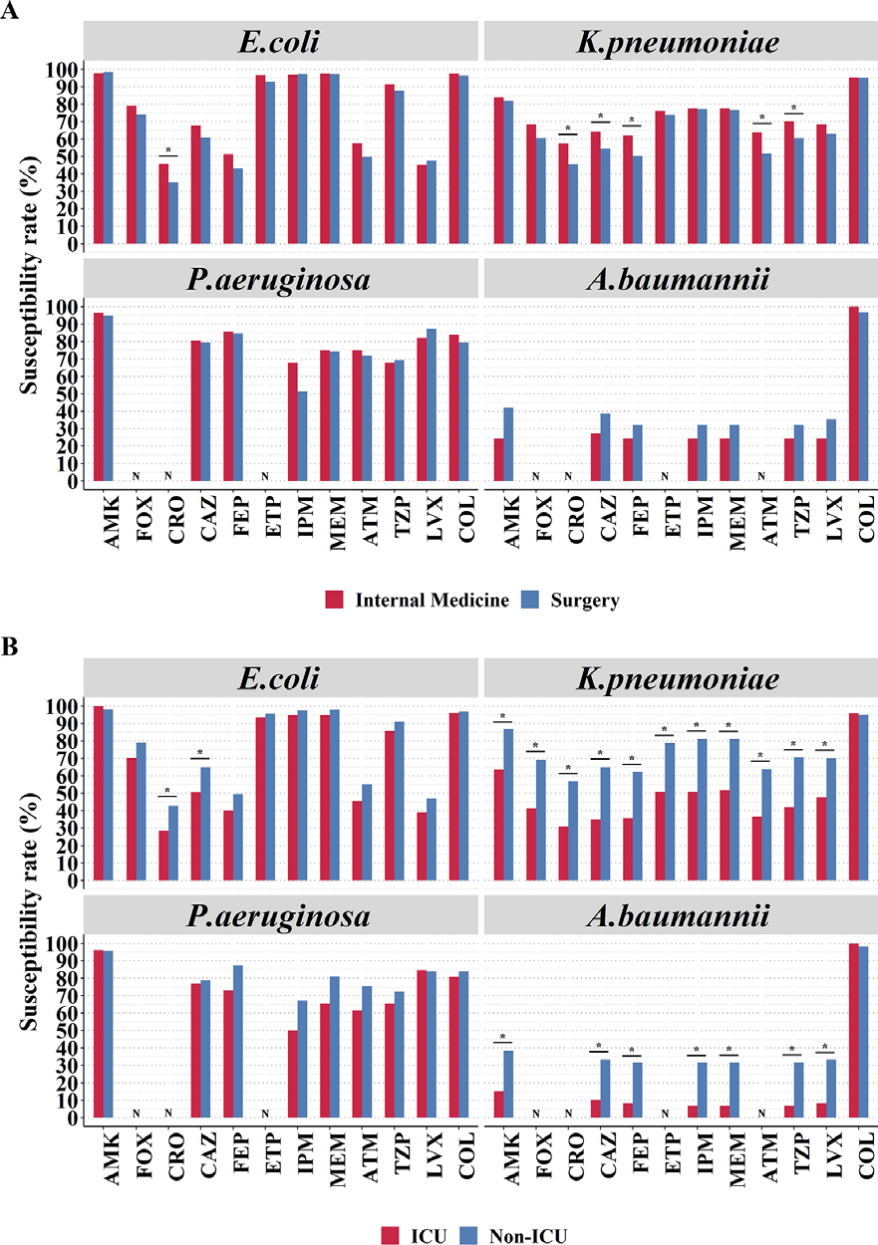
Comparison of susceptibility rates of common antimicrobials to *E. coli*, *Klebsiella pneumoniae, P. aeruginosa*, and *Acinetobacter baumannii* in (A) Internal Medicine and Surgery; (B) ICU and non-ICU departments. Note: *: *P* < 0.05, *χ*^2^ test followed by post hoc Fisher’s exact test with Bonferroni correction. Abbreviations: N, Not tested; AMK, amikacin; ATM, aztreonam; CAZ, ceftazidime; COL, colistin; CRO, ceftriaxone; ETP, ertapenem; FEP, cefepime; FOX, cefoxitin; IPM, imipenem; LVX, levofloxacin; MEM, meropenem; TZP, piperacillin-tazobactam.

When comparing internal medicine and surgery, *E. coli* exhibited a significantly higher susceptibility to ceftriaxone in internal medicine compared to surgery (45.7% vs. 35.2%, *P* = 0.011) departments. In internal medicine departments, *K. pneumoniae* showed significantly higher susceptibilities to various antimicrobials, including ceftriaxone (57.6% vs. 45.5%, *P* = 0.017), ceftazidime (64.3% vs. 54.5%, *P* = 0.047), cefepime (62.2% vs. 50.3%, *P* = 0.017), aztreonam (63.9% vs. 51.5%, *P* = 0.013), and piperacillin-tazobactam (70.2% vs. 60.5%, *P* = 0.042), compared to surgery departments (all *P* < 0.05) (Figure 1).

In the comparison between ICU and non-ICU departments, *K. pneumoniae* had significantly lower susceptibility rates to the various antibiotics tested including amikacin (63.5% vs. 87.0%), cefoxitin (41.3% vs. 69.3%), ceftriaxone (31.0% vs. 56.8%), ceftazidime (34.9% vs. 65.0%), cefepime (35.7% vs. 62.4%), ertapenem (50.8% vs. 78.8%), imipenem (50.8% vs. 81.1%), meropenem (51.6% vs. 81.1%), aztreonam (36.5% vs. 63.7%), piperacillin-tazobactam (42.1% vs. 70.6%), and levofloxacin (47.6% vs. 70.1%) in ICU departments compared to non-ICU departments (all *P* < 0.05). Similarly, *A. baumannii* exhibited significantly lower susceptibility rates to aminoglycosides, third-generation cephalosporins, carbapenems, β-lactamase inhibitor compounds, and fluoroquinolones in ICU departments (6.8–15.3%) compared to non-ICU departments (31.7–38.3%) (all *P* < 0.05) (Figure 1).

### Comparison of the detection rates of CR strains and ESBL-producing strains by age groups and regions in China

**Figure 2.**
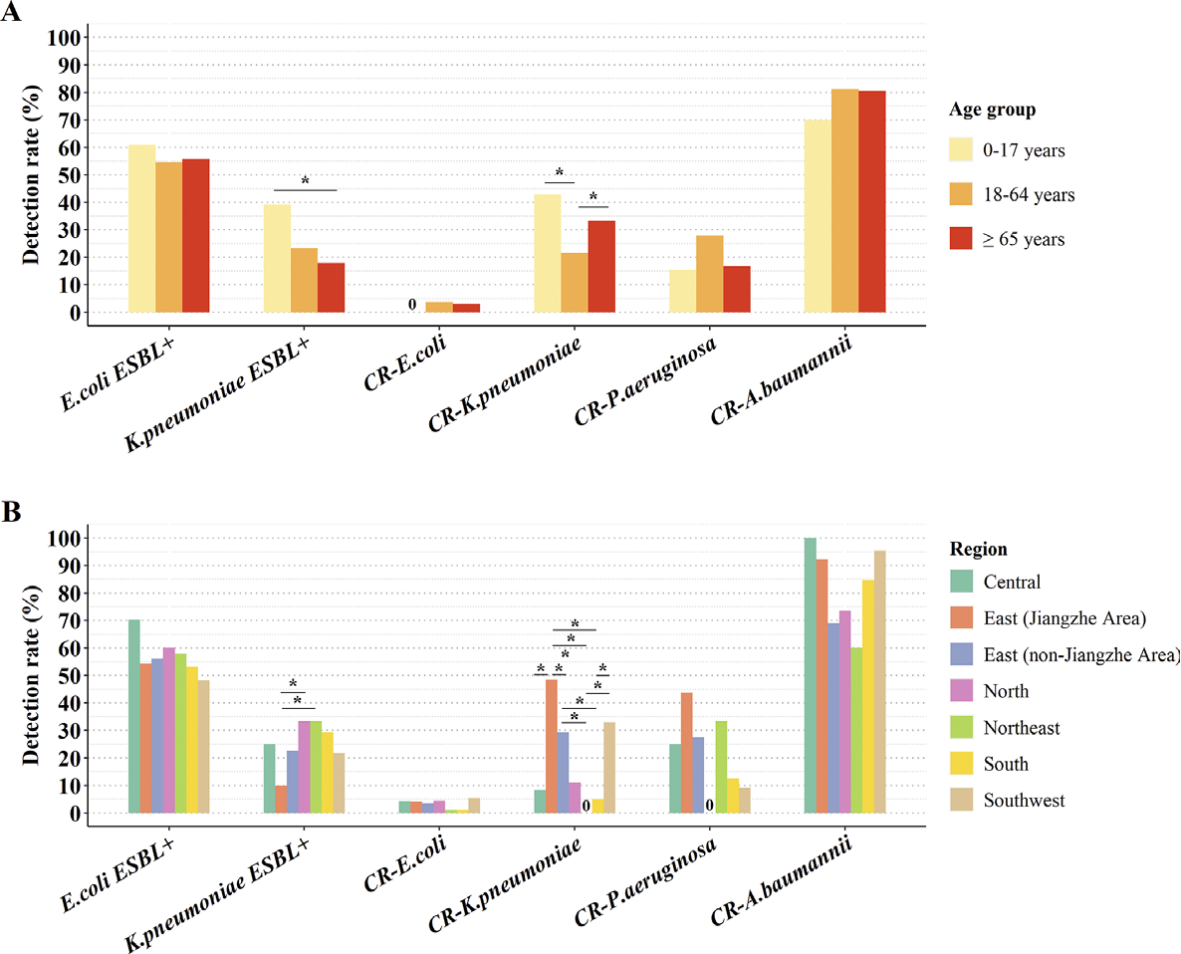
Comparison of detection rates of carbapenem-resistant and ESBL-producing strains in different (A) age groups and (B) regions of China. Note: *:*P* < 0.05, *χ*^2^ test followed by a post hoc Fisher’s exact test with Bonferroni correction. Abbreviations: 0, the detection rate was 0.0%; CR, carbapenem-resistant; *E. coli* ESBL+, ESBL-producing *Escherichia coli*; *Klebsiella pneumoniae* ESBL+, ESBL-producing *K. pneumoniae.*

The detection rates of CRKP varied significantly in different age groups (*P* = 0.003). Notably, the rate for CRKP was higher in children and adolescents aged 0–17 years (42.9% vs. 21.6%) and in the elderly aged ≥65 years (33.3% vs. 21.6%) compared to the group aged 18–64 years (*P* < 0.05). Additionally, there were significant differences in the detection rate of CRKP in different regions of China (*P* < 0.001). Specifically, the detection rate of CRKP in the East (Jiangzhe Area) (48.5%) was significantly higher than in the East (non-Jiangzhe Area) (29.4%), North (11.1%), Central (8.3%), South (4.9%), and Northeast (0.0%) regions (all *P* < 0.05) (Figure 2).

There was a significant difference in the detection rate of *K. pneumoniae* ESBL+ among various age groups (*P* = 0.030). The detection rate of *K. pneumoniae* ESBL+ in minors aged 0–17 years was significantly higher compared to those aged ≥65 years (39.3% vs. 17.9%) (*P* < 0.05). Additionally, there was also a variation in the detection rate of *K. pneumoniae* ESBL+ across different regions of China (*P* = 0.016). The detection rate of *K. pneumoniae* ESBL+ in the East (Jiangzhe area) (9.9%) was significantly lower than in the North (33.3%) and Northeast (33.3%) regions (both *P* < 0.05) (Figure 2).

## Discussion

This study presents the distribution characteristics of GNB from BSI in different years, age groups, and regions of China from 2018 to 2020. In terms of antimicrobial susceptibility analysis, the susceptibility rates of *K. pneumoniae* and *A. baumannii* in ICU departments were significantly lower than those in non-ICU departments. In addition, the detection rates of CRKP and *K. pneumoniae* ESBL+ varied significantly across different age groups and regions, which is an important finding in this nationwide antimicrobial resistance surveillance for BSI.

*E. coli* and *K. pneumoniae* are the main GNB-causing BSI for all the age groups with boundaries of 18 and 65 years of age, and the proportion of *E. coli* appeared to increase with the age group, which was the same distribution features of Gram-negative bacteria bloodstream infections reported by the China Bloodstream Gram-negative Pathogens Antimicrobial Resistance and Virulence Surveillance Network (CARVIS-NET) study (1,939 isolates from 21 hospitals from 2019 to 2021) [12]. However, the prevalence of *K. pneumoniae* ESBL+ (22.2% vs. 32.5%) appeared to be notably lower than the national BRICS during the earlier period (2014–2019) [4]. This finding may be attributed to a declining trend in *K. pneumoniae* ESBL+ incidence over the years in China [4]. Additionally, all of the hospitals that participated in this study were large tertiary hospitals, with 74% situated in economically developed regions such as the eastern, central, northern, and southern regions of China. National BRICS data indicated a significantly lower prevalence of *K. pneumoniae* ESBL+ in developed regions compared to developing regions of China [4].

Regarding the distribution of GNB in different departments, it was found that *A. baumannii* was more common in ICU patients than in non-ICU patients (17.5% vs. 4.1%), while *E. coli* was more common in non-ICU cases than in ICU cases (22.8% vs. 47.0%). A multicenter study on bloodstream pathogen distribution and resistance monitoring in Italy also found that patients in medical wards had a higher chance of being infected with *E. coli* compared to ICU patients (OR = 5.37), while the probabilities for *K. pneumoniae* (OR = 0.49) and *A. baumannii* (OR = 0.24) infections were lower [9]. It is clear that medical interventions can influence the spectrum of pathogens in patients with BSI.

In recent years, there has been a growing trend of BSIs caused by *A. baumannii*, making it a focal point of concern for hospital-acquired infections [13]. Particularly, alarming is the high rate of carbapenem resistance exhibited by *A. baumannii.* The mechanisms underlying CR *A. baumannii* (CRAB) include intrinsic and acquired β-lactamases, upregulation of efflux pumps, decreased outer membrane permeability, and alterations in the antibiotic targets [14-16]. Additionally, patterns of antimicrobial usage significantly influenced the development of CRAB resistance [17]. In 2013, among 208 hospitals in China, CRAB accounted for 53.5% of blood isolates, far exceeding other GNB. In the present study, 80.0% (96/120) of *A. baumannii* were CR, and the susceptibility rates of *A. baumannii* to antibiotics other than colistin were < 27.5%. It is worth noting that the resistance of *A. baumannii* was more severe in ICU departments. A survey of 77 ICUs in various provinces of China also found a prevalence rate of 71.4% for CRAB [18]. Due to the high mortality rate and poor prognosis associated with CRAB infections [19], coupled with the limited treatment options available to intensive care physicians, it is essential to prioritize monitoring the use of colistin and tigecycline and regular surveillance of their resistance levels. This approach will be crucial to maintain the effectiveness of last-resort antibiotics in clinical settings characterized by high levels of resistance [20].

In China, CRKP has become a major concern in the clinic, with the China antimicrobial surveillance network (CHINET) report revealing that it was caused by 72.4% of carbapenem-resistant *Enterobacterales* (CRE) infections in 2019 [21]. The BRICS report further highlighted the rising prevalence of CRKP from 7.0% in 2014 to 19.6% in 2019 [4]. BSI caused by CRKP is particularly alarming, as they are associated with a high mortality rate of 42–84% [22]. The present study analyzed the detection rate of CRKP based on age and regions in China and has produced significant guidance for clinical empirical therapy and policymaking. The detection rate of CRKP was higher in patients aged 0–17 years and in patients aged ≥65 years. The Infectious Disease Surveillance of Pediatrics (ISPED) programme reported that the proportion of CRKP in Chinese children from 2016 to 2020 was 19.7% [23]. The surveillance programme also revealed a gradual decrease in CRKP prevalence with increasing age, posing a potential threat to newborn infants [23]. Newborns and paediatric patients face an increased risk of CRKP infection due to their immature immune systems [24]. The high detection rate of CRKP in patients aged ≥65 years may be related to the high morbidity and mortality rate of BSI in the elderly population [25], as well as their greater risk of contracting a Gram-negative bacterial infection exhibiting antimicrobial resistance [26]. CRKP infection is an independent risk factor for the 90-day mortality rate in elderly patients with *K. pneumoniae* infection, while prior use of carbapenems increased the risk of CRKP infection in this population [27]. In clinical practice, implementing active surveillance cultures in high-risk units, particularly neonatal and geriatric wards, is crucial for the early detection of CRKP colonization. Additionally, the antimicrobial susceptibility results from our study suggest that empirical therapy for suspected CRKP infections could include colistin, pending definitive susceptibility results. The present study also highlights the importance of considering regional factors when studying BSI caused by GNB. The detection rate of CRKP in the East (Jiangzhe region) was significantly higher than in most other regions, and *K. pneumoniae* accounted for the highest proportion of GNB (44.3%) in the East (Jiangzhe region), surpassing other regions. The China CRE Network also reported significant regional differences in the incidence of CRE infections, with the highest incidence being in Jiangsu province [28]. The possible reasons for these outcomes may be that the eastern region is more economically developed in China, leading to a higher density of medical facilities, population, increased pressure on antibiotic usage and more frequent medical procedures and interventions. In China, CRKP primarily acquires resistance through the production of carbapenemases [29, 30]. However, the epidemiological characteristics and resistance mechanisms of CRKP strains vary across different regions, and these variations are closely associated with patients’ clinical outcomes [31-33]. This phenomenon underscores the importance of conducting region-specific epidemiological investigations.

The limitations of the present study include the inability to identify factors associated with resistance patterns, due to the absence of detailed patient information or basic characteristics data. Moreover, selection biases may have occurred since the numbers of screened GNB isolates delivered from each region of China differed and may not reflect the situation of the entire populations in the respective regions. Additionally, the annual variability in the number of collected isolates, notably the reduced number in 2020 potentially attributable to the COVID-19 pandemic, may have had an impact on the consistency of the data and consequently affected the interpretation of temporal trends. Finally, the local epidemiological context might restrict the generalizability of our findings.

## Conclusion

*E. coli* and *K. pneumoniae* were found to be the leading causes of BSI. The severity of *A. baumannii* drug resistance, particularly in ICU departments, underscores the need to prioritize monitoring colistin usage and to regularly monitor its susceptibility in settings with high resistance levels. The high detection rates of CRKP in the eastern region of China, as well as among the elderly and underage populations, emphasize the importance of resistant pathogens and prudent antibiotic selection in economically developed regions and vulnerable populations. Overall, the present study has provided valuable surveillance data on the epidemiology of GNB-causing BSI in China, with important implications for guiding antimicrobial drug selection and formulating prevention and control strategies for these infections.

## Supporting information

Chen et al. supplementary material Chen et al. supplementary material

## Supplementary material

For supplementary material accompanying this paper visit http://doi.org/10.1017/S0950268824001286. click here to view supplementary material

## Future directions

Building upon these findings, in addition to ongoing epidemiological investigations, further studies are needed to explore the molecular mechanisms of resistance in both *K. pneumoniae* and *A. baumannii*, as well as to evaluate the impact of intervention measures on resistance patterns.
