# Supplementary material for: Antimicrobial susceptibility of gram-negative strains isolated from bloodstream infections in China: Results from the study for monitoring antimicrobial resistance trends (SMART) 2018–2020
Source: Epidemiol Infect. 2025 Mar 21;153:e48. doi: 10.1017/S0950268824001286 (PMC11951230; doi:10.1017/S0950268824001286)
Supplement: Chen et al. supplementary material [file S0950268824001286sup001.docx]

***Epidemiology and Infection***

**Antimicrobial susceptibility of Gram-negative strains isolated from bloodstream infections in China:** **results from the Study for Monitoring Antimicrobial Resistance Trends (SMART) 2018 – 2020**

Yili Chen^1,#^, Pingjuan Liu^1,#^, Huayin Li^2^, Wenxiang Huang^3^, Chunxia Yang^4^, Mei Kang^5^, Xiaofeng Jiang^6^, Bin Shan^7^, Hong He^8^, Fuping Hu^9^, Pengcheng Li^10^, Yingchun Xu^11,*^, Kang Liao^1,*^

^1^Department of Laboratory Medicine, The First Affiliated Hospital, Sun Yat-Sen University, Guangzhou 510080, China; ^2^Division of Microbiology, Zhongshan Hospital of Fudan University, Shanghai 200032, China; ^3^Division of Microbiology, The First Affiliated Hospital of Chongqing Medical University, Chongqing 400016, China; ^4^Department of Clinical Laboratory, Beijing Chao-Yang Hospital, Beijing 100043, China; ^5^Department of Laboratory Medicine, West China School of Medicine, West China Hospital of Sichuan University, Chengdu 611130, China; ^6^Department of Clinical Laboratory, The Fourth Affiliated Hospital of Harbin Medical University, Harbin 150001, China; ^7^Department of Clinical Laboratory, First Affiliated Hospital of Kunming Medical University, Kunming 650032, China; ^8^Department of Clinical Laboratory, The Affiliated Hospital of Qingdao University, Qingdao 266000, China; ^9^Institute of Antibiotics, Huashan Hospital, Fudan University, Shanghai 200040, China; ^10^V&I, Global Medical & Scientific Affairs, MSD China, Shanghai 200233, China; ^11^Division of Microbiology, Peking Union Medical College Hospital, Peking Union Medical College, Chinese Academy of Medical Sciences, Beijing 100730, China

^#^These authors contributed equally to this work.

***Correspondence to:**

**Yingchun Xu**

Division of Microbiology, Peking Union Medical College Hospital, Peking Union Medical College, Chinese Academy of Medical Sciences, No. 1 Shuaifuyuan, Wangfujing Street, Beijing 100730, China

Tel: +86-010-69159766

Email: xycpumch@139.com

and

**Kang Liao**

Department of Laboratory Medicine, The First Affiliated Hospital, Sun Yat-Sen University, No. 58 Zhongshan 2nd Road, Guangzhou 510080, China

Tel: +86-020-87332200-8461

Email: liaokang1971@163.com

**Supplementary Table 1.** The 18 participating hospitals and their regions in China

| Regions | Participation hospitals |
| --- | --- |
| Central | Tongji Medical School of Mid-China |
| East (Jiangzhe Area) | 1st Affiliated Hospital of Zhejiang University Medical College |
|  | 1st Affiliated Hospital of Zhejiang University |
|  | Jinling Hospital, Nanjing University |
| East (non-Jiangzhe Area) | 2nd Affiliated Hospital of Nanchang University |
|  | Provincial Hospital Shandong University |
|  | Shanghai Huashan Hospital |
|  | The Affiliated Hospital of Medical College, Qingdao University |
|  | Zhongshan Hospital of Fudan University |
| North | Beijing Chao-yang Hospital |
|  | Peking Union Medical College Hospital |
| Northeast | Jilin Province Peoples Hospital |
|  | The Fourth Affiliated Hospital of Harbin Medical University |
| South | 1st Affiliated Hospital of Sun Yat-Sen University |
|  | Haikou Peoples Hospital of Central South University |
| Southwest | Chongqing Medical University Affiliate No. 1 Hospital |
|  | No. 1 Affiliated Hospital of Kunming Medical University |
|  | West China School of Medicine/West China Hospital of Sichuan |


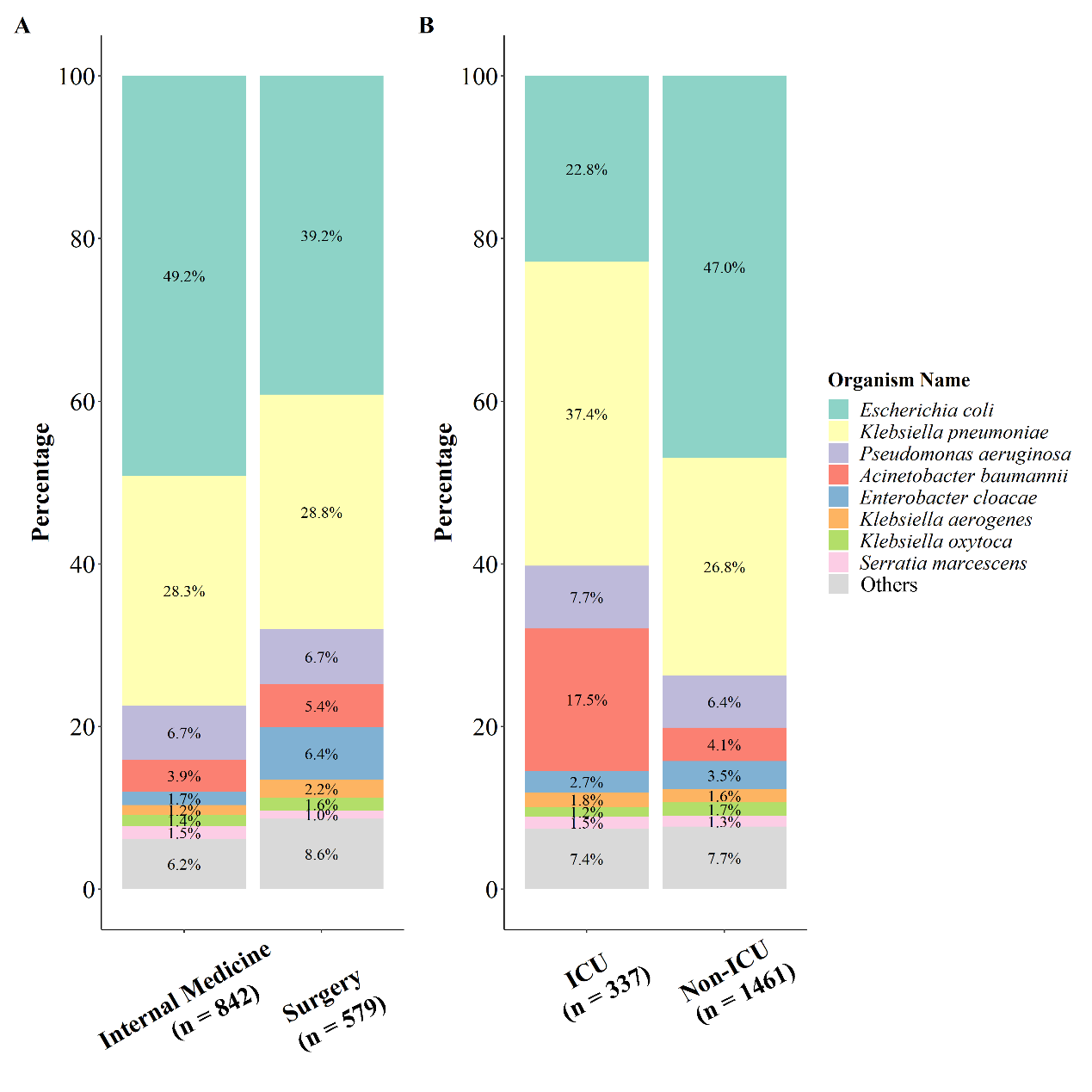


**Supplementary Figure 1.** Species distribution of 1,815 isolates of Gram-negative bacilli collected from blood stream infections in A) Internal Medicine and Surgery; B) ICU and non-ICU departments: SMART 2018 – 2020, China.


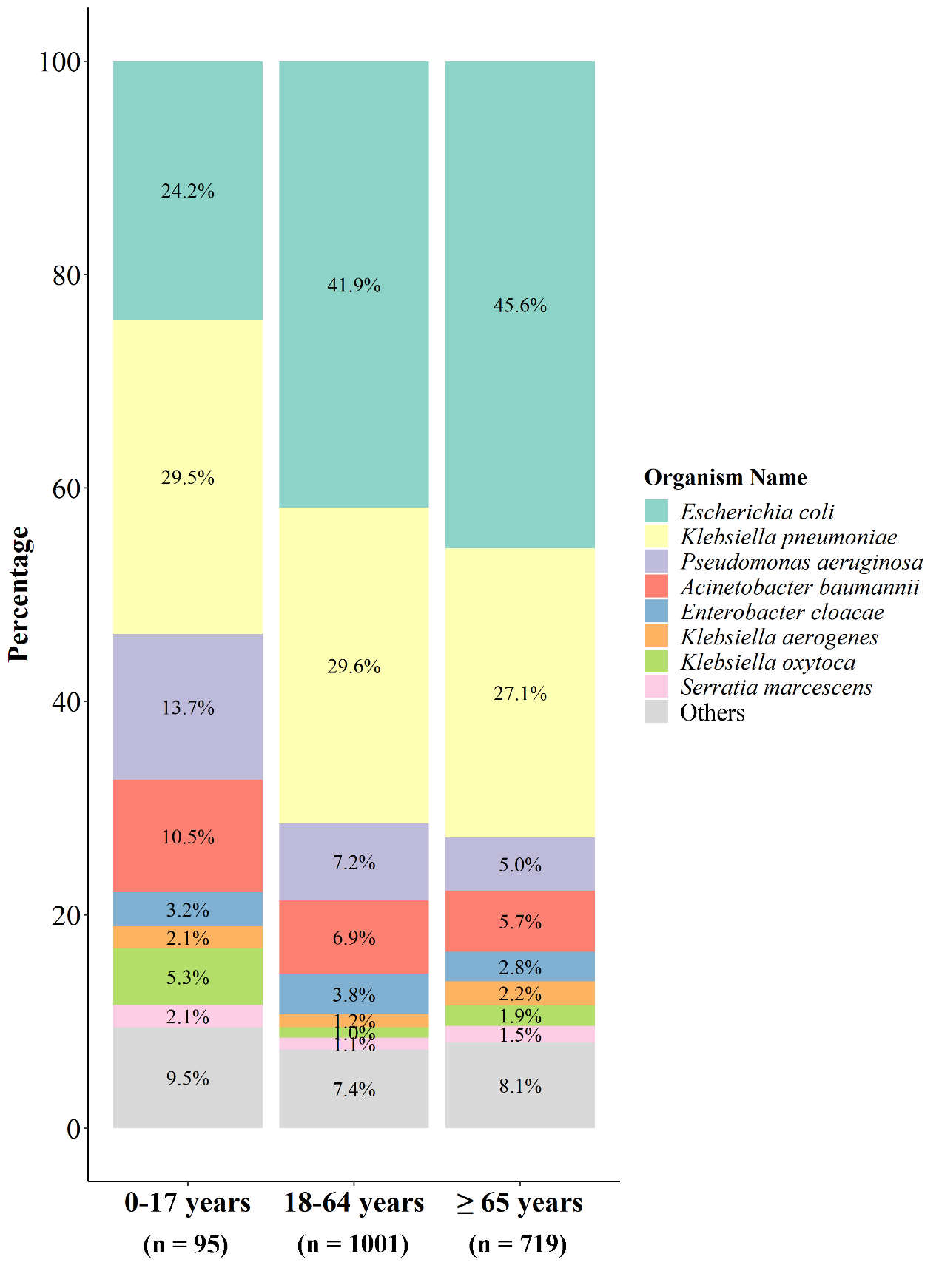


**Supplementary Figure 2.** Species distribution of 1,815 isolates of Gram-negative bacilli collected from blood stream infections in different age groups: SMART 2018 – 2020, China.


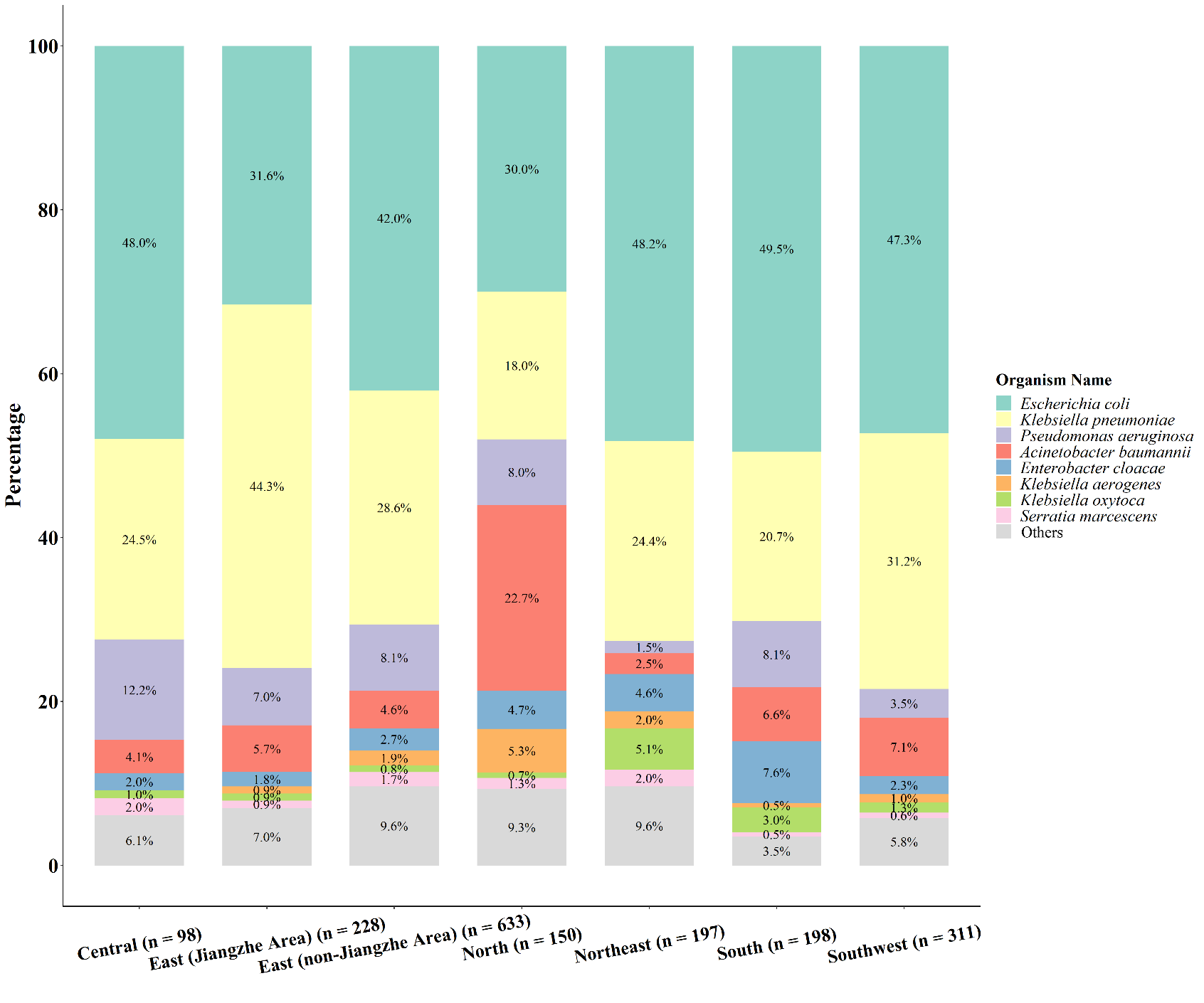


**Supplementary Figure 3.** Species distribution of 1,815 isolates of Gram-negative bacilli collected from blood stream infections in different regions: SMART 2018 – 2020, China.
